# Extracellular Vesicles in Osteosarcoma: Antagonists or Therapeutic Agents?

**DOI:** 10.3390/ijms222212586

**Published:** 2021-11-22

**Authors:** Viviana De Martino, Michela Rossi, Giulia Battafarano, Jessica Pepe, Salvatore Minisola, Andrea Del Fattore

**Affiliations:** 1 Department of Clinical, Internal, Anaesthesiology and Cardiovascular Sciences, Sapienza University, 00185 Rome, Italy; Viviana.demartino@uniroma1.it (V.D.M.); jessica.pepe@uniroma1.it (J.P.); Salvatore.minisola@uniroma1.it (S.M.); 2 Bone Physiopathology Research Unit, Genetics and Rare Diseases Research Division, Bambino Gesù Children’s Hospital, IRCCS, 00165 Rome, Italy; michela1.rossi@opbg.net (M.R.); giulia.battafarano@opbg.net (G.B.)

**Keywords:** osteosarcoma, extracellular vesicles, therapy

## Abstract

Osteosarcoma (OS) is a skeletal tumor affecting mainly children and adolescents. The presence of distance metastasis is frequent and it is localized preferentially to the lung, representing the main reason for death among patients. The therapeutic approaches are based on surgery and chemotherapeutics. However, the drug resistance and the side effects associated with the chemotherapy require the identification of new therapeutic approaches. The understanding of the complex biological scenario of the osteosarcoma will open the way for the identification of new targets for its treatment. Recently, a great interest of scientific community is for extracellular vesicles (EVs), that are released in the tumor microenvironment and are important regulators of tumor proliferation and the metastatic process. At the same time, circulating extracellular vesicles can be exploited as diagnostic and prognostic biomarkers, and they can be loaded with drugs as a new therapeutic approach for osteosarcoma patients. Thus, the characterization of OS-related EVs could represent a way to convert these vesicles from antagonists for human health into therapeutic and/or diagnostic agents.

## 1. Introduction

Osteosarcoma (OS) is a highly malignant skeletal tumor characterized by the presence of neoplastic cells of mesenchymal origin that deposit an immature osteoid matrix. Despite its rarity, osteosarcoma is the third most frequent primary malignancy affecting mainly children, adolescents and young adults [1]. Osteosarcoma is more common in male individuals, with an overall ratio between males and females of 1.43:1. The incidence is 2–3 cases per 1,000,000 inhabitants/year [1]. The peak incidence occurs in the group from 10 to 19 years old and seems to be related to the period in which maximum bone growth occurs, suggesting a connection between tumor formation and growth factors expressed during bone growth. A second peak of OS in adults over 65 years of age has been reported [2,3].

The most affected areas are the metaphyses of long bones and bone segments such as the proximal tibia, distal femur, proximal humerus, and all areas characterized by a massive bone rearrangement; it rarely occurs in flat bones and spine [4]. The most common symptom in patients is a relatively non-specific pain in the affected area, often wrongly attributed to bone growth, accompanied by swelling of soft tissues. The manifestation of pain can result from the weakening of the bone with the development of microfractures; severe pain occurs in case of more serious pathological fractures, found in more than 15% of pediatric patients. Symptoms of general malaise, including weight loss, pallor, fever and/or anorexia are very rare [4].

Several subtypes of osteosarcoma can be identified: classical intramedullary or central (osteoblastic, chondroblastic and fibroblastic); telangiectasic; small cell; high-grade surface; secondary osteosarcoma; parosteal; periostal; and central with a low degree of malignancy. The first type of OS (classical intramedullary or central) is the most common among teenagers and includes about 85% of all OS cases [5].

Osteosarcoma is characterized by highly invasive ability. The presence of distant metastases is very frequent and represents the main reason of death among osteosarcoma patients; the preferential site of metastasis is the lung [6,7]. The 5-year survival rate of OS patients with metastasis is 20% compared to 65% of patients with localized disease [4,8]. Consequently, due to its aggressiveness and the lowest overall survival rates, metastatic osteosarcoma is considered one of the main causes of death.

Numerous studies have been carried out on the cytogenetic and molecular aspects of osteosarcoma with often conflicting results; therefore, their diagnostic and prognostic value still appears limited. The rarity and the heterogeneity of the pathology also do not help to clarify its etiological meaning. Osteosarcoma is counted among complex karyotype sarcomas [9]. Seventy percent of osteosarcoma cases show numerical, structural alterations and genomic amplifications. Cytogenetic analysis revealed numerous breaking points and translocations, underlining the complexity and instability of the genetic background in this tumor [10]. At the molecular level, the most compromised signaling pathways are linked to the altered activity of oncogenes, such as Myc (avian myelocytomatosis viral oncogene homolog) and tumor suppressors genes (Rb (retinoblastoma protein) and p53), which are functionally inactivated in most cases of osteosarcoma [11]. Recently, genomic alterations in genes involved in the mechanisms of DNA repair were reported in a subset of patients exhibiting a specific combination of single-base substitutions, LOH (loss of heterozygosity), or large-scale genome instability signatures characteristic of BRCA1/2-deficient tumors [12].

Although osteosarcoma is sporadic in 95% of cases, several inherited syndromes such as Li–Fraumeni, Rothmund–Thomson and Retinoblastoma familial cancers have been associated with a predisposition to develop osteosarcoma [13,14,15]. Paget’s disease, generally a benign condition characterized an increase in bone turnover, could represent a risk condition for osteosarcoma [16]. Chronic osteomyelitis, osteochondroma, encondroma and fibrous dysplasia are also associated with osteosarcoma [2,11].

For a diagnosis, a set of clinical analyses, radiological investigations and the evaluation of the pathological tissue by performing biopsy is required [17].

Currently, the therapeutic strategies for osteosarcoma include three treatments: the surgical approach, and neoadjuvant and adjuvant chemotherapy [18,19]. Indeed, about 85% of cases of high-grade OS can be successfully resected and reconstructed, preserving the affected limb and its function [20]. A meta-analysis performed by Xiaojuan Li et al. reported that patients subjected to limb salvage surgery (LSS) had a similar local recurrence compared to patients treated with amputation [21]. In addition, they found that the 5-year overall survival rate of patients treated with LSS was higher than those treated with amputation [22]. Amputation is generally reserved only for those tumors in which a complete resection of tumor and the preservation of limb function is not feasible [23]. Neoadjuvant chemotherapy is administered about 8–10 weeks before surgery; the use of preoperative chemotherapy offers time for surgical planning, decreases tumor size and potentially facilitates its removal, reduces the risk of distant metastases and allows assessment of response to therapy [20]. The intensification of neoadjuvant chemotherapy increased the number of good respondents but did not alter overall survival [21,24]. Today, cooperative group studies in North America and Europe provided a standard protocol neoadjuvant chemotherapy, known as MAP, characterized by the use of multi-drugs such as methotrexate in high doses (HDMTX), doxorubicin (adriamycin, ADM) and cisplatin (CDP) [25]. Numerous clinical trials have tested various combinations of the five chemotherapeutic agents known to be active in this disease (methotrexate, doxorubicin, cisplatin, ifosfamide and etoposide) [26,27]. Although the chemotherapy has improved the life of osteosarcoma patients, the onset of drug resistance, toxicity and related side effects limits the use of these chemotherapy agents in clinical practice [28,29].

The identification of new therapeutic targets is therefore necessary above all in patients who have chemoresistance or who experience local relapses (35% of patients) or lung metastases (60% of patients) [4]. The development of chemoresistance induces complications, linked above all to the therapeutic need to increase the dose of drug for treatment, which is not always well tolerated by the patient due to its high toxicity, and often to stop treatment [30,31]. In the past few years, there is increased attention on understanding the complex biological scenario in osteosarcoma. Due to the inter- and intratumoral heterogeneity, a unique targeted pattern does not exist, and this has made attempts unsuccessful over the past three decades [32]. The development of novel therapeutic strategies remains an important clinical need. In this review, we summarized the new advances in osteosarcoma biology, particularly the involvement of extracellular vesicles as potential diagnostic and prognostic biomarkers and as a new therapeutic approach for osteosarcoma.

## 2. Molecular Mechanisms of Osteosarcoma Progression

Osteosarcoma develops in the bone microenvironment, a very specialized environment in which bone cells (Mesenchymal stem cells (MSC), osteoblasts, osteocytes, osteoclasts precursors and osteoclasts), and immune and vascular cells communicate with each other to maintain the integrity of the skeleton [2,33]. This is a soil rich in growth factors, cytokines, chemokines and extracellular vesicles that create a fertile microenvironment for osteosarcoma growth [34].

Alterations of the bone remodeling are the first steps in the osteosarcoma onset. In the past 15 years, osteosarcoma was commonly described as a disease related to the alterations of MSC; recently, it was demonstrated that osteosarcoma can also occur following dysregulation of multiple points in bone development [35].

Regarding the role of MSC in the osteosarcoma progression, two different MSC populations exist in the osteosarcoma microenvironment. Naïve MSC derive from normal tissue and can exert pro- and antitumoral activity [36,37]; the tight crosstalk between MSC and osteosarcoma cells leads to the reprogramming of MSC into MSC stimulating tumor progression (tumor-tissue educated MSC) [38].

Indeed, osteosarcoma cells can modulate the microenvironment; the high-rate energetic glycolytic metabolism of cancer cells causes high lactic acid production and a high proton efflux; short-term acidosis activates downstream signaling of the NF-kB (Nuclear factor-kappa B) pathway in MSC but not in osteosarcoma cells [39]. Low extracellular pH in these tumors induces an increased invasive behavior and promotes the secretion of high levels of Interleukin 6 (IL-6) and IL-8 by mesenchymal stem cells, stimulating osteosarcoma growth and metastasis [39]. IL-8 can activate the chemokine receptor CXCR1 (C-X-C Motif Chemokine Receptor 1) and can lead to anoikis resistance of osteosarcoma cells and progression of pulmonary metastasis. Furthermore, MSC also secretes CCL5 (C-C motif ligand 5), SDF-1 (Stromal derived factor 1) and VEGF (Vascular Endothelial Growth Factor), promoting osteosarcoma progression, angiogenesis and metastasis [39,40].

In vivo experiments revealed that OSDC (Osteosarcoma associated stromal cells, also named osteosarcoma-derived cells) and MSC co-injections with tumor cells led to increased tumor growth and eventually to metastases in nude and/or severe combined immunodeficiency (SCID) mice [41].

Acidosis, hypoxia and inflammation induce neovascularization that allows the delivery of nutrients and oxygen to the tumor cells. In the tumor microenvironment, tumor cells and endothelial cells express pro-angiogenic factors as VEGF, PDGF (Platelet Derived Growth Factor), FGF (Fibroblast Growth Factor) and TGF-β (Transforming Growth Factor beta) [42].

Osteosarcoma is a highly vascularized bone tumor and mainly occurs in the region of bone growth close to metaphysis, where type-H endothelial cells promoting angiogenesis are located, suggesting their role in osteosarcoma neo-angiogenesis [43,44]. The neo-angiogenesis in osteosarcoma could derive from pre-existing vessels or be dependent from endothelial progenitor cells that can differentiate into mature endothelial cells [45]. However, vascular mimicry is also observed in osteosarcoma and is characterized by the formation of vasculogenic-like microchannels generated by tumor cells; this alternative process of angiogenesis and vasculogenesis occurs in about 20% of patients and is associated with poor prognosis [46,47]. The mechanisms of vascular mimicry still remain largely unknown; however, the autocrine VEGF/VEGFR1 (VEGF Receptor 1) signaling has been proposed as key pathway for the vasculogenic features of osteosarcoma cells, and a clear correlation between VEGF levels and tumor progression was demonstrated [48,49].

Factors secreted in the bone microenvironment also contribute to the abnormal osteoclast activity. At the same time, osteoclast bone resorption can lead to the release of pro-tumor factors as IGF-1 (Insulin Growth Factor 1) and TGF-β from the bone matrix that stimulate tumor cells [50,51]. The role of osteoclasts in the onset and progression of osteosarcoma is still controversial [52,53]. There are clinical and experimental data showing that the presence of osteoclasts in osteosarcoma-adjacent tissue is associated with poor outcomes [52], but on the other hand, published studies suggest that the presence of osteoclasts at the primary site of OS lesions prevents metastasis [52,54]. Endo-Munoz et al. found that expression of osteoclast-specific tartrate-resistant acid phosphatase 5 (TRAcP5) is significantly downregulated in biopsies isolated from osteosarcoma patients compared with nonmalignant bone tissue. However, the lesions of patients with lung metastasis had increased levels of TRAcP5 expression compared to lesions of non-metastatic disease [53].

Understanding the role of osteoclasts in osteosarcoma onset, progression and metastasis is relevant for therapeutic approaches. If osteoclasts are essential for the lung metastasis [53], the administration of antiresorptive drugs including bisphosphonates and Denosumab would be effective therapeutic strategy; if the bone resorption suppresses the metastasis development, this approach would be contraindicated. A phase III clinical trial study demonstrated a worse therapeutic outcome following a combined treatment with chemotherapy/surgery and antiresorptive zoledronic acid [55].

Tumor cells also express MMP-9 (Matrix Metallopeptidase-9) that allows the dissemination of tumor cells and, at the same time, is essential for the activation of angiogenic factors [7].

Regarding the role of immune cells in the progression of osteosarcoma, both myeloid and lymphoid cells have been detected in osteosarcoma [56]. Particularly, tumor-associated macrophages (TAM) were detected in osteosarcoma biopsies, and they were associated with reduced metastasis and improved survival by still-unclear mechanisms [2,57,58]. Moreover, osteosarcoma tumors are characterized by poor infiltration of CD8^+^ lymphocytes, suggesting the poor immunogenic feature of this tumor; the scarce infiltrate of cytotoxic lymphocytes allowed the osteosarcoma to be defined as cold tumor [59]. Moreover, the ratio between CD8^+^ cells and regulatory T cells (CD4^+^FoxP3^+^) in bone biopsies from osteosarcoma patients is important for discriminating between patients with an expected prolonged survival from those with a poor prognosis [60]. The presence of Antigen Presenting Cells (APC), including dendritic cells and CD68^+^ macrophages, has been associated with a poorer prognosis [61].

From this general overview, it is quite evident that in the osteosarcoma microenvironment there is a tight crosstalk among bone, endothelial and immune cells, mediated by cell-cell contact, soluble factors and extracellular vesicles. Indeed, it was demonstrated that EVs are spontaneously released by osteosarcoma cells in the microenvironment and they can exert several functions: they can mediate the immune escape of tumor cells, and promote angiogenesis, proliferation and metastatic activity of osteosarcoma cells [62].

## 3. Extracellular Vesicles

EVs are lipid-bound vesicles secreted by cells into the extracellular space [63,64]. Extracellular vesicles can be vehicles for nucleic acids (DNA, RNA and microRNAs (miRNAs)), proteins, lipids (eicosanoids, fatty acids and cholesterol), and also intact organelles [63]. It was reported that EVs can contain mitochondria that can be transferred from the parent/donor to recipient cells [65].

**Figure 1:**
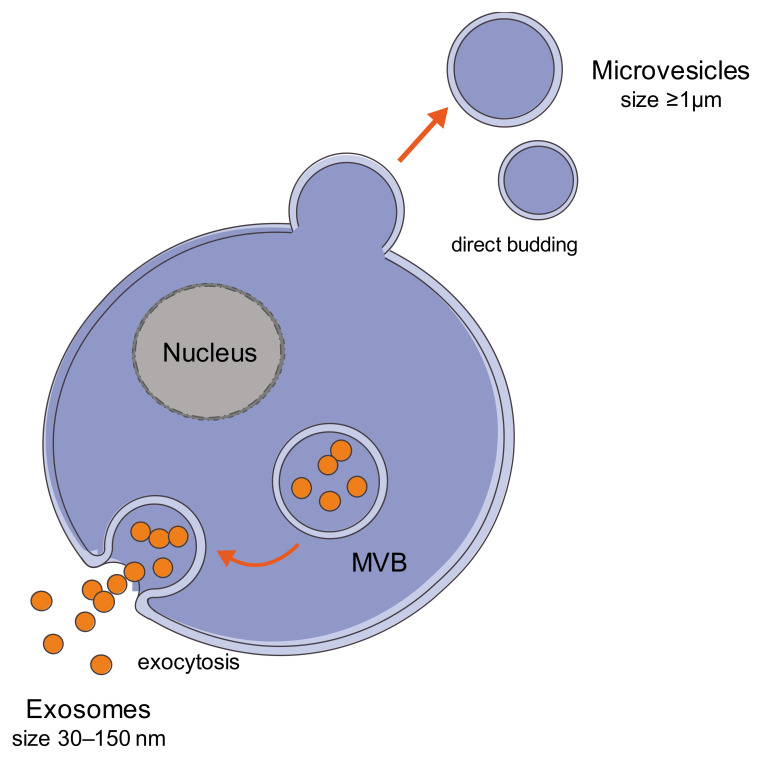
Extracellular vesicles (EVs). EVs represent a heterogeneous population of vesicles, including microvesicles and exosomes, differing in size, content and biogenesis. Microvesicles (up to 1 µm) are produced by direct outward budding of the cell membrane; exosomes are small vesicles (30–150 nm) and are released by fusion of multivesicular bodies (MVBs) with the plasma membrane into the extracellular space. Figure created using Servier Medical Art (https://smart.servier.com; accessed on 1 October 2021).

They represent a heterogeneous population of vesicles, including microvesicles and exosomes, differing in size, content and biogenesis [66,67]. Exosomes are vesicles typically 30–150 nm in diameter and are produced by inward budding of the limiting membrane of early endosomes, which mature into multivesicular bodies (MVBs) during the process [64,68]. MVB contains small vesicles, and its fusion with plasma membrane can allow the secretion of exosomes into the extracellular space. Microvesicles have a diameter up to 1 µm, and they are produced by direct outward budding of the cell membrane; the exact mechanisms of microvesicle production are not completely understood; however, they involve the cytoskeleton components and the fusion machinery [67,68] (Figure 1).

No specific protein markers have been identified to distinguish the different types of EVs [69]. However, substantial overlap of protein profiles is often observed, due in part to the lack of standardized isolation and analysis methods of EVs.

Recent published studies suggest that EVs can be used as a prognostic/diagnostic tool for several diseases and as a therapeutic approach [70,71,72,73,74]. At the same time, it was demonstrated that cancer cells can use EVs as a mechanism to expulse chemotherapy drugs, contributing to drug resistance [75,76].

### 3.1. Role of EVs in Osteosarcoma Microenvironment and Tumoral Growth

In 2013, Garimella et al. reported the presence of extracellular vesicles in the osteosarcoma microenvironment of an OS orthotopic mouse (BOOM) model using a human OS cell line 143B [77]. Electron microscopic examination revealed the presence of EVs of 50–200 nm in diameter that derive from bone and tumor cells. MSC-derived exosomes can promote cell proliferation, migration and invasion in osteosarcoma in vitro and in vivo [78,79]. Moreover, MSC-EVs can also promote autophagy contributing to MSC-EV-induced promotion of malignant tumorigenesis [78].

Moreover, multivesicular bodies were detected in the tumor tissue. A great interest of the scientific community regards the identification of the proteins delivered by these extracellular vesicles [77]. Mannerström et al. demonstrated that OS-EVs modulate the epigenetic status of MSC through hypomethylation of long interspersed nuclear element 1 [80]. It was demonstrated that EVs secreted by the osteosarcoma 143B cell line contain a pro-osteoclastogenic cargo, which includes MMPs (MMP-1 and MMP-13), RANK-L (Receptor Activator of Nuclear Factor κ B Ligand), CD-9 and TGF-β [81], involved in the bone remodeling activity.

Indeed MMP-1 (Matrix metalloproteinase-1) and MMP-13 (Matrix metalloproteinase-3) are the key collagenases responsible for degradation of type I collagen [82]. They are important in regulating the osteoblastic differentiation and also bone erosion [82]. The significantly higher expression of MMPs and downregulation of the MMP13-targeting miRNA143 (miR-143) are related to poor prognostic outcomes in patients with OS [83].

RANK-L is the main osteoclastogenic cytokine that binds to the RANK receptor expressed on osteoclast precursors and osteoclasts. The RANK-L/RANK binding stimulates osteoclast differentiation and bone erosion; at the same time, it was demonstrated that extracellular vesicles can also carry RANK, and its binding to RANK-L expressed on osteoblast membrane stimulates the bone formation [2]. The presence of RANK on extracellular vesicles isolated from osteosarcoma cells still needs to be evaluated.

CD9 belongs to the Tetraspan transmembrane (TM4)-superfamily proteins and can be associated with integrins, heparin-binding EGF-like growth factor, small G proteins, and other TM4-superfamily proteins, CD63, CD81 and CD82 [84,85,86]. Its expression on stromal cell is essential for osteoclastogenesis [87]. CD9 is also important for the bone metastasis since it was reported that CD9 was significantly overexpressed in bone metastases versus primary tumors and visceral metastatic lesions, and that its inhibition delays homing of tumor cells in bone marrow, slowing down bone destruction [88].

The delivery of TGF-β by EV is important for the regulation of bone remodeling; indeed, this growth factor is mainly released from the bone matrix during the bone resorption activity, and it is able to stimulate osteoblast progenitors and osteosarcoma cells, and to regulate osteoclastogenesis [81,89].

Moreover, Raimondi et al. showed that OS-derived EVs stimulated endothelial cells to express and secrete elevated levels of the proangiogenic factor VEGF and interleukins (IL-6 and IL-8) [90].

### 3.2. Role of EVs in Osteosarcoma Metastasis

The establishment of cancer metastasis involves several steps, including intravasation, arrest at a distant organ, extravasation and growth in secondary sites [91]. The mechanisms involved in the migration arrest of metastatic cells are still controversial [92]. Over 80% of all metastases in OS occur in the lung [93]; the mechanism by which osteosarcoma cells prefer lung to metastasize is still under investigation. Several papers suggested that the C-X-C-motif chemokine receptor 4 (CXCR4) and the interaction with CXCL12 ligand play a relevant role in this process. Indeed, CXCL12 is expressed at high levels in the lung, and CXCR4 expression was high in osteosarcoma-patient samples [7,94]. Another possible mechanism is mediated by CXCR3 expressed in osteosarcoma and CXCL9-10-11 expressed in the lung [95]; the interaction led to the increase of proliferation and invasion of tumor cells in the metastatic organ [7,96].

The genesis of a metastasis requires the adhesion of cancer cells to the new environment; a crucial role in this step is played by ezrin that is linked to Rho and PI3K/Akt pathways [97,98].

Several reports suggest that EVs released by tumor cells regulate the metastatic process [99]. Indeed, it was demonstrated that tumor-derived exosomes can directly stimulate the metastasis and can regulate the microenvironment to support tumor growth [100]. Mazumdar et al. reported how osteosarcoma-derived EVs could influence the differentiation of lung fibroblast into a cancer-associated fibroblast supporting metastatic progression [101]. OS-derived EVs may furthermore contribute to the metastatic process by prompting MSC to acquire a pro-tumorigenic and pro-metastatic phenotype [102,103]. Moreover, exosomes released by osteosarcoma contain urokinase plasminogen activator (uPA) [104]; interestingly, the autocrine and paracrine activation of the uPA/uPAR axis has been related to the conversion of OS cells to a metastatic phenotype [104].

Macklin et al. showed that EVs secreted by cells derived from a highly metastatic clonal variant of osteosarcoma can be internalized by a poorly metastatic clonal variant and induced a migratory and invasive phenotype [105]. In addition, it was demonstrated that EVs released by highly metastatic clones selectively concentrate within lung tissue where they may set up a chemotactic gradient to recruit osteosarcoma cells to the pre-metastatic niche within the lung [105].

It was suggested that miRNAs contained in the exosomes can have a crucial role in the metastasis [106,107]. Jerez et al. isolated miRNAs from extracellular vesicles released from six different human osteosarcoma or osteoblastic cell lines with different degrees of metastatic potential (i.e., SAOS2, MG63, HOS, 143B, U2OS and hFOB1.19). About 300 miRNAs are contained in EVs of each cell line, and about 70 are expressed at high level. MiR-21-5p (microRNA-21-5p), miR-143-3p, miR-148a-3p and miR-181a-5p are relatively abundant in vesicles from metastatic cells compared to the non-metastatic MG63 [108]. MiR-21 is already well described as oncomir [47]. Regarding the role of miR-143-3p, some studies suggested that it may counteract metastatic properties of squamous cell carcinoma [109]. MiR-148a-3p and miR-181a-5p have been detected in serum samples from gastrointestinal cancer patients [110,111]. However, bioinformatics analysis revealed that these miRNAs can regulate apoptosis, angiogenesis, cell adhesion and migration [108].

There is abundant evidence that long ncRNA (lncRNA) also plays a key role in the development and progression of OS [112]. In particular, the study by Li et al. disclosed that highly enriched lncRNA OIP5-AS1 in exosomes secreted by OS cells regulates angiogenesis and ATG5-mediated autophagy in OS through miR-153, thereby participating in the formation and development of malignant tumors [113].

TGF-β can regulate tumor invasion, metastasis, angiogenesis and cell apoptosis [114,115,116]. TGF-β has been detected in exosomes released by osteosarcoma cells, and this can influence the metastasis process [117]. Indeed, TGF-β can regulate the secretion of CXCL16 by osteoclasts that stimulate osteoblastic and osteosarcoma cell migration, regulating the metastatic process [118]. Moreover, vesicular TGF-β induces proinflammatory IL-6 production by MSCs, allowing the tumor EV-educated mesenchymal stem cells to promote osteosarcoma progression together with intratumor STAT3 activation and lung metastasis formation [119]. Indeed, the IL-6/STAT3 signaling pathway stimulates cell proliferation and migration/invasion, and protects tumor cells from drug-induced apoptosis [120].

### 3.3. Role of EVs in Chemioresistance

Exosomes can be used by tumor cells to develop chemoresistance [75]. In vitro and in vivo studies linked EVs to drug resistance in many tumors, including osteosarcoma [121,122,123]. Indeed, EVs can represent a way to eliminate apoptotic stimuli from the cells or can acquire antitumor drug substance to achieve resistance, for example, to paclitaxel [124,125]. Torreggiani et al. showed that exosomes derived from doxorubicin-resistant osteosarcoma cells could be taken up into secondary cells, thus inducing a doxorubicin-resistant phenotype. Moreover, they suggested that the mechanism by which exosomes transfer drug resistance among osteosarcoma cells is mediated by multidrug resistance-associated protein 1 (MDR-1) mRNA and P-glycoprotein [122].

Accordingly, Pan et al. demonstrated that exosomes derived from cisplatin-resistant osteosarcoma cells reduce the sensitivity of MG63 and U2OS cells to cisplatin, inhibit apoptosis, and increase the expression of MDR-1 and P-glycoprotein [126]. Moreover they revealed a relationship between the levels of circular_103801 miRNA carried by exosomes in patients’ sera and their survival, suggesting circulating exosomes and miRNA as a prognostic tool [126].

Xu et al. demonstrated that a substantial profile of exosomal miRNAs, including miR-124, miR-133a, miR-135b, miR-148a, miR-199a-3p, miR-27a, miR-385, and miR-9, was dysregulated in poor chemotherapeutic response [127].

These results would assist with potential clinical chemotherapeutic treatment of OS and help in monitoring or predicting disease progression during chemotherapy in osteosarcoma.

### 3.4. Role of EVs in Therapy

Due to the rarity of osteosarcoma, several anatomical-clinical variants, genome complexity, different presentation modalities of disease and age of the affected population, the treatment of this disease still remains an unsolved problem [11]. Indeed, in the past 40 years, few changes have been reported for clinical care of patients.

A recent study of Kyung-Mi and coauthors revealed that EVs exert an anti-tumoral effect on osteosarcoma cells. EVs from canine macrophages activate apoptotic pathways in canine OS cells and can be an effective anticancer therapeutic approach [128]. Moreover, MSC-derived EVs carrying miR-150 reduce proliferation and migration of osteosarcoma cells by targeting IGF2BP1 (Insulin-like Growth Factor-2 mRNA-Binding Protein 1) [129].

**Figure 2:**
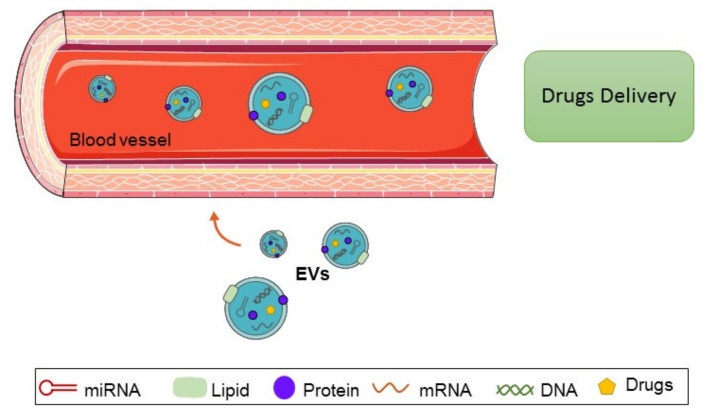
Extracellular vesicles as a vehicle for delivering chemotherapeutic drugs. Figure created using Servier Medical Art (https://smart.servier.com; accessed on 1 October 2021).

Several studies suggest that exosomes can be used as a vehicle to deliver chemotherapeutic drugs to osteosarcoma cells (Figure 2) [122,130,131]. Indeed, it was demonstrated that exosomes can be directly charged with drugs [132,133]; another approach is to load the cells with drugs that will be removed from the cells via extracellular vesicles.

Several papers suggested that mesenchymal stem/stromal cells isolated from the bone marrow can be loaded with chemotherapic drugs, and exosomes isolated from the conditioned medium exert a pro-apoptotic effect on tumor cells [134,135]. Furthermore, MSC can be loaded with synthetic miRNAs that can be transferred into recipient cells and suppress migration of OS cells [136,137].

In some cases, the apoptotic effect was increased following the treatment with drug EVs compared to that observed with a free drug at the same concentration contained in the EVs [37], confirming that EV itself can exert an apoptotic stimulus [128].

### 3.5. EVs for Diagnosis

Extracellular vesicles can be detected in body fluids, including blood, urine and cerebrospinal fluid, and great interest is for their use as a diagnostic and/or prognostic tool [69,138,139,140]. Indeed the presence of membrane structure provides stability and allows prolonged periods of storage of EVs before analysis, making their clinical use feasible [141]. EVs are highly produced by tumoral cells compared to healthy cells, and are usually present at increased levels at tumor diagnosis and/or can increase during tumor progression [142]. EV cargo reflects metastatic progression and treatment response [143,144]. Xu et al. deciphered alteration of specific miRNAs in patients with a poor chemotherapeutic response when compared with good responders [127]. A recent and promising study by Cambier and colleagues reported the possibility to use OS-associated EVs as possible liquid biopsies for early detection of cancer. The authors identified in OS patients’ EV-specific repetitive element DNA sequences compared to a control serum EV preparation [145].

The major concern regarding the study on EVs is the lack of standardization protocols for their isolation and analysis [146,147]. Indeed, many methods are reported to isolate EVs, including ultracentrifugation, filtration, sucrose gradient and mixed protocols [148,149,150]. Another problem is related to their quantification [151]. Many studies were conducted performing a quantification by FACS (fluorescence activated cell sorting) analysis [152]. However, the limit of instrument resolution is usually about 0.5 µm, making the quantification not appropriate with this method; the other approach is based on NTA (Nanoparticle Tracking Analysis) technology that allows visualization and measurement of nanoparticles in suspension in the range of 10–1000 nm based on the analysis of Brownian motion [153]. However, a great standardization protocol is required [69], and in this respect the International Society of Extracellular Vesicles supported several initiatives, such as the EV Transparent Reporting and Centralizing Knowledge [154], the Minimal Information for Studies of EVs [147] and the Clinical Wrap-Up session at ISEV2018 [155].

## 4. Conclusions

There is a need for therapeutic approaches to improve the survival of patients with a poor response. Indeed, there are many challenges to face. First of all, osteosarcoma is a rare disease with approximately 400 patients diagnosed every year in the United States, making it difficult to complete an accurate clinical trial. Moreover, some of the molecular mechanisms, including TP53 or Rb, altered in osteosarcoma are difficult to target.

The leading cause of mortality in osteosarcoma patients continues to be the development of metastasis; understanding the biology of osteosarcoma and the role of extracellular vesicles will open the way for developing or identifying novel therapeutics to prevent or arrest metastasis. In the future we believe that new diagnostic and therapeutic approaches could exploit the same mechanisms, including extracellular vesicles that cancer cells use to grow and disseminate, for the establishment of more specific treatments for patients. The identification of molecules carried by OS-derived EVs that help tumor growth, survival and metastasis could represent a way to convert these antagonists into a therapeutic and/or diagnostic tool. However, a major limitation in this field is the lack of a well-standardized method for EV isolation and quantification, and a rigorous methodology and characterization will open the avenue for to better employ EVs as therapeutic carriers and diagnostics tools.
